# Mixed Pain: More Than the Sum of Its Parts?

**DOI:** 10.1002/ejp.70430

**Published:** 2026-10-16

**Authors:** Jan D. Wandrey, Jan Vollert

**Affiliations:** ^1^ Department of Clinical and Biomedical Sciences, Faculty of Health and Life Sciences University of Exeter Exeter UK; ^2^ Charité – Universitätsmedizin Berlin, Corporate Member of Freie Universität Berlin and Humboldt‐Universität zu Berlin, Department of Anaesthesiology and Intensive Care Campus Charité Mitte and Campus Virchow‐Klinikum Berlin Germany

This journal recently published a paper by Freynhagen et al., entitled Mixed Pain: Toward a Consensus Definition and a Mechanism‐Based Framework (Freynhagen et al. 2026).

Classification is a fundamental piece of research: without classifying subject matter, one cannot construct models, formulate explanations or make predictions (Khalidi 2023). Yet, whether a classification captures meaningful distinctions remains open to empirical investigation. Conceptual frameworks can therefore be understood as lenses or lighthouses: they illuminate selected aspects of complex phenomena while inevitably leaving others less visible (Bordage 2009). From this perspective, the value of the proposed mixed pain framework presented by Freynhagen et al. (Freynhagen et al. 2026) may lie in providing a common classification through which we can study mixed pain and build an empirical foundation whether it is a distinct clinical phenotype.

The distinction between nociceptive, neuropathic, and nociplastic pain has substantially advanced pain classification and enabled specialised treatment pathways, with entire research fields evolving from such terms. Yet, these conceptual distinctions do not always translate into clearly circumscribed categories in clinical practice. As outlined in the manuscript, patients frequently present with features crossing their boundaries, and conditions such as chronic low back pain, postsurgical pain, osteoarthritis, or cancer pain may not fit neatly within a single mechanistic pain descriptor (Freynhagen et al. 2026). This is particularly apparent for nociplastic pain. Its clinical criteria allow nociceptive or neuropathic contributions to coexist, if these do not entirely explain the pain.

The proposed definition of mixed pain explicitly represents such overlap, encompassing the four different combinations of nociceptive, neuropathic, and nociplastic pain, or all three of them (Freynhagen et al. 2026). The important question is what these combinations represent. Are they merely a description of coexisting pain components, or does their coexistence carry information beyond the individual components—in other words, can the entirety be more than the sum of its parts?

The consensus framework makes this question accessible to systematic investigation. The defined combinations can be studied for differences in clinical presentation, prognosis, and treatment response, and for interactions that cannot be explained by their individual components alone. Whether mixed pain ultimately represents a clinically meaningful classification can therefore be tested rather than assumed.

This is particularly important for the development and testing of treatment options. Pharmacological approaches already cross the boundaries of mechanistic pain descriptors. Gabapentinoids, for example, are established treatments for neuropathic pain but are also used in fibromyalgia, commonly considered the prototypical nociplastic pain condition. Although pharmacological response is not a specific marker of pain mechanism, this overlap suggests that treatment effects may extend across mechanistic pain descriptors and that these descriptors may not be as clearly separated as their classification implies. Other pharmacological interventions, and most if not all non‐pharmacological interventions, can be thought to address multiple mechanisms. A standardised mixed pain framework could enable studies investigating whether combinations of descriptors predict differential responses to pharmacological, non‐pharmacological or multimodal interventions.

The crucial next step is therefore empirical. Prospective studies should investigate whether the proposed combinations differ from their respective individual components in natural history, prognosis, or treatment response. Importantly, analyses should determine whether the coexistence or interaction of components provides information beyond their additive effects. Such studies could establish whether identifying mixed pain improves prediction or treatment selection beyond identifying its individual components alone. They could also determine whether the four combinations represent clinically meaningful phenotypes, heterogeneous manifestations of a broader concept, or simply convenient descriptions of coexisting pain types.

A scientific classification does not need to provide the final answer to be useful. Indeed, classification can be what makes the relevant questions accessible to systematic investigation in the first place (Khalidi 2023). The proposed mixed pain framework should therefore perhaps be regarded neither as proof of a new pain phenotype nor as redundant simply because its individual components can already be described. Its immediate contribution is to provide a common language for investigating their coexistence. Whether mixed pain ultimately proves to be more than the sum of its parts is an empirical question, but one that may be considerably easier to answer once we have agreed on what we mean by it.

## Funding

J.D.W. was a participant in the BIH Charité Digital Clinician Scientist Program funded by the DFG, the Charité—Universitätsmedizin Berlin, and the Berlin Institute of Health at Charité (BIH). J.V. was supported by the National Institute for Health and Care Research (NIHR) Exeter Biomedical Research Centre (BRC).

## Conflicts of Interest

The authors declare no conflicts of interest.

## Linked Articles

This article is linked to Freynhagen et al., Kosek, Attal, and Freynhagen papers. To view these articles, visit https://doi.org/10.1002/ejp.70356, https://doi.org/10.1002/ejp.70386, https://doi.org/10.1002/ejp.70379 and https://doi.org/10.1002/ejp.70415.
